# Contacts to general practice in the 12 months preceding a diagnosis of an abdominal cancer: a national register-based cohort study

**DOI:** 10.1080/02813432.2022.2057054

**Published:** 2022-04-01

**Authors:** Nanna Holt Jessen, Henry Jensen, Alina Zalounina Falborg, Henning Glerup, Henning Gronbaek, Peter Vedsted

**Affiliations:** a Research Unit for General Practice, Research Centre for Cancer Diagnosis in Primary Care, Aarhus, Denmark; b Department of Public Health, Aarhus University, Aarhus, Denmark; c Department of Clinical Medicine, Diagnostic Center, Silkeborg Regional Hospital, Aarhus University, Silkeborg, Denmark; d Department of Hepatology and Gastroenterology, Aarhus University Hospital, Aarhus, Denmark

**Keywords:** Abdomen, Denmark, general practice, neoplasms, population, primary health care, referral and consultation

## Abstract

**Objective:**

To compare the number of contacts to general practice across 11 types of abdominal cancer in the 12 months preceding a diagnosis.

**Design:**

Nationwide register study.

**Setting:**

Danish general practice.

**Subjects:**

Forty-seven thousand eight hundred and ninety-eight patients diagnosed with oesophageal, gastric, colon, rectal, liver, gall bladder/biliary tract, pancreatic, endometrial, ovarian, kidney or bladder cancer in 2014–2018.

**Main outcome measures:**

Monthly contact rates and incidence rate ratios (IRRs) of daytime face-to-face, email and telephone consultations in general practice across different abdominal cancers. The analyses were conducted for each sex and adjusted for age, comorbidity, marital status and education.

**Results:**

Compared to women with colon cancer, women with rectal cancer had the lowest number of contacts to general practice (IRR 12 months pre-diagnostic (IRR_–12_)=0.86 (95% CI: 0.80–0.92); IRR 1 month pre-diagnostic (IRR_–1_)=0.85 (95% CI: 0.81–0.89)), whereas women with liver (IRR_–12_=1.23 (95% CI: 1.09–1.38); IRR_–1_=1.11 (95% CI: 1.02–1.20)), pancreatic (IRR_–12_=1.08 (95% CI: 1.01–1.16); IRR_1_=1.52 (95% CI: 1.45–1.58)) and kidney cancer (IRR_–12_=1.14 (95% CI: 1.05–1.23); IRR_–1_=1.18 (95% CI: 1.12–1.24)) had the highest number of contacts. Men showed similar patterns. From seven months pre-diagnostic, an increase in contacts to general practice was seen in bladder cancer patients, particularly women, compared to colon cancer.

**Conclusions:**

Using pre-diagnostic contact rates unveiled that liver, pancreatic, kidney and bladder cancers had a higher and more prolonged use of general practice. This may suggest missed opportunities of diagnosing cancer. Thus, pre-diagnostic contact rates may indicate symptoms and signs for cancer that need further research to ensure early cancer diagnosis.

**Key points**
- The majority of cancer patients attend their general practitioner (GP) before diagnosis; however, little is known about the use of general practice across different abdominal cancers.
- This study suggests that a potential exists to detect some abdominal cancers at an earlier point in time.
- The contact patterns in general practice seem to be shaped by the degree of diagnostic difficulty.
- GPs may need additional diagnostic opportunities to identify abdominal cancer in symptomatic patients.

## Introduction

General practitioners (GPs) act as gatekeepers to the rest of the healthcare system in Denmark, except for emergencies, otorhinolaryngologists and ophthalmologists. Thus, most cancer patients attend their GP prior to a diagnosis of cancer [1,2]. The GPs are responsible for referring patients to relevant diagnostic investigations in case of suspicion of cancer. Abdominal symptoms account for approximately 10% of encounters in general practice [3,4], and many symptoms and signs of abdominal cancer mimic each other [4,5]. Therefore, it can be difficult to identify the abdominal cancer causing the presented symptoms [6], and the GP may thus not take appropriate actions, which may lead to missed opportunities for more timely diagnosis of the cancer [7].

Missed opportunities are instances in which post hoc judgement indicates that alternative decisions or actions could have led to a more timely diagnosis, and such opportunities may occur in different phases of the diagnostic process [7]. Thus, delayed diagnosis may be attributable to a missed opportunity [2]. As shorter time to diagnosis is associated with a more favourable prognosis in several types of cancers [8], identifying possibilities to diagnose the cancer at an earlier point in time is an important step to shorten the time to diagnosis, including the time spent in general practice [9,10].

Studies indicate that increased use of healthcare services can be seen as a proxy for presentation of symptoms [11,12], and an increased frequency of healthcare utilisation among cancer patients is seen already several months before their diagnosis compared to patients without cancer [1,13,14]. This increase may represent missed opportunities, indicating opportunities to diagnose some cancers at an earlier point in time. This diagnostic window may vary across different types of abdominal cancers.

It has been suggested that cancers can be categorised into easy-to-diagnose, intermediate-to-diagnose and hard-to-diagnose cancer types according to how difficult the cancer is to suspect and diagnose in general practice; this categorisation is based on the consultation frequency in the year preceding a diagnosis [15]. However, the timing of these consultations is unclear across different abdominal cancers.

We aimed to compare the frequency and timing of contacts to general practice across different abdominal cancer types in the 12 months preceding a diagnosis, to identify possible missed opportunities to diagnose the cancer at an earlier point in time.

## Methods

### Study design

We conducted a national registry-based cohort study of first-time abdominal cancer patients, who were diagnosed between 1 January 2014 and 31 December 2018. We used the unique Danish civil registration number, which is assigned to all Danish residents at birth or immigration [16], to link the data at an individual level across registers.

### Setting

The study was set in Denmark, where a publicly funded healthcare system offers free access to both general practice and hospital care. Almost all Danish residents (>98%) are registered with a specific general practice [17], which they must consult for medical advice.

### Study population

Patients eligible for inclusion were first-time cancer patients, aged ≥18 years, registered in the Danish Cancer Registry (DCR) [16] with an abdominal cancer in Denmark in the study period, and with no prior history of cancer (except prior non-melanoma skin cancer). Patients with a valid civil registration number, patients who had lived in Denmark during (at least) the 12 months preceding the date of diagnosis, and patients listed with a general practice were included.

The included abdominal cancer types were defined according to the International Classification of Disease (ICD-10), version 2016, and comprised the following cancer types: oesophageal (C15.0–C15.9), gastric (C16.0–C16.9), colon (C18.0–C18.9), rectal (C20), liver (C22.0–C22.9), gall bladder/biliary tract (C23 and C24.0–C24.9), pancreatic (C25.0–C25.9), endometrial (C54.0–C54.9 and C55), ovarian (C56), kidney (C64 and C65) and bladder cancer (C67.0–C67.9). The date of diagnosis and the ICD-10 diagnosis code were retrieved from the DCR.

### Outcomes

The main outcome was number of contacts to general practice. This number included daytime face-to-face consultations (consultations in general practice and home visits), telephone consultations and email consultations. Telephone and email consultations were included because consultations in general practice are increasingly conducted through different communication technologies in Denmark [18]. However, differences in symptom presentation are seen across abdominal cancers [5,19], and these differences may also be reflected in the mode of contact used by abdominal cancer patients to contact general practice. Therefore, a sub-analysis was performed to explore whether differences were observed in the type of contact to general practice across the abdominal cancers.

Data were obtained from the Danish National Health Service Register, which holds information on all types of consultations involving general practice [17]. The data are registered electronically as part of remuneration system.

### Covariates

To adjust for differences between groups we included: sex, age, comorbidity, marital status and educational level. Age at the date of diagnosis was modelled through restricted cubic splines with three knots according to Harrell’s recommended percentiles [20]. The Charlson Comorbidity Index (CCI) was calculated based on diagnoses registered in the DNPR in the 10 years preceding study entry (i.e. starting from one year before the date of the cancer diagnosis) and grouped into ‘none’ (CCI score: 0), ‘moderate’ (CCI score: 1–2) and ‘severe’ (CCI score: ≥3). Marital status and educational level were collected from Statistics Denmark [16]. Marital status was categorised into ‘living alone’ and ‘married/cohabitating.’ The highest attained level of education was categorised according to the International Standard Classification of Education (ISCED) into ‘low’ (ISCED level: 1–2), ‘medium’ (ISCED level: 3–4) and ‘high’ (ISCED level: ≥5). Missing information on educational level was seen in 2% of the patients and was recoded as ‘low’ as prior studies have shown that these patients often have lower levels of education [21].

### Statistical analysis

Monthly mean contact rates in general practice were calculated for each of the 12 months preceding the date of diagnosis. All patients were included in the analyses and used in the denominator of the rate calculation during all 12 months prior to the diagnosis. The point of increase was defined as the first month prior to diagnosis when the rates were higher than in the month immediately before, and this point was determined by the corresponding confidence intervals (CIs) (i.e. the CIs for two adjacent months did not overlap).

Generalised linear models with log link for the negative binomial family were used to calculate incidence rate ratios (IRRs) to enable comparison of monthly rates of contacts to general practice between different abdominal cancer types. Colon cancer was chosen as the reference cancer because this cancer type has been suggested to belong to the intermediate-to-diagnose group of cancers [15], and is the most common abdominal cancer type in both sexes [22]. Cluster robust variance estimation was applied to account for possible cluster effects at patient level as measurements on the same person were repeated monthly. IRRs were adjusted for age, comorbidity, marital status and educational level. All analyses were performed for each sex separately, as differences in healthcare use have previously been demonstrated to depend on sex [1,23].

The sub-analysis assessed the monthly contact rates and the IRRs for the different abdominal cancersstratified by type of contact (daytime face-to-face consultation, telephone consultation or email consultation) in general practice.

A *p* value of ≤.05 was considered statistically significant, and estimates were provided with a 95% CI. Analyses were performed using Stata statistical software, version 15 (StataCorp LP, College Station, TX).

## Results

A total of 47,898 abdominal cancer patients were included, of which colon cancer patients constituted 31.4%. Women represented 47.0% of the cases; the mean age was 69.5 years (standard deviation (SD)=12.2), 30.5% had comorbidity, and 43.9% had a lower level of education. Men were slightly younger, had more comorbidity, and had higher levels of education than women (Table 1).

**Table 1.** Characteristics of the study population shown by abdominal cancer type and total and stratified by sex (*N* = 47,898).

	Oesophageal		Gastric		Colon		Rectal		Liver		Gall bladder/ biliary tract		Pancreatic		Endometrial		Ovarian		Kidney		Bladder		Total	
	Men	Women	Men	Women	Men	Women	Men	Women	Men	Women	Men	Women	Men	Women	Men	Women	Men	Women	Men	Women	Men	Women	Men	Women
Number, *n* (%)	1685 (6.6)	578 (2.6)	1795 (7.1)	865 (3.8)	7857 (30.9)	7160 (31.8)	4449 (17.5)	2727 (12.1)	1432 (5.6)	596 (2.6)	411 (1.6)	495 (2.2)	2227 (8.8)	2077 (9.2)	n/a	3517 (15.6)	n/a	2002 (8.9)	2750 (10.8)	1474 (6.5)	2788 (11.0)	1013 (4.5)	25,394 (100.0)	22,504 (100.0)
Age, mean (SD)	68.3 (10.1)	70.7 (10.8)	68.4 (11.8)	69.3 (12.9)	69.9 (10.8)	71.1 (12.1)	68.3 (10.6)	68.6 (12.4)	68.8 (10.3)	70.1 (12.5)	69.6 (11.6)	71.5 (10.7)	68.9 (10.4)	71.3 (11.6)	n/a	67.6 (11.4)	n/a	66.1 (13.3)	64.8 (11.4)	66.8 (12.2)	72.4 (10.0)	72.4 (10.8)	69.0 (10.9)	69.5 (12.2)
Age group, *n* (%)																								
Age <65 years	581 (34.5)	168 (29.1)	617 (34.4)	273 (31.6)	2077 (26.4)	1813 (25.3)	1467 (33.0)	992 (36.4)	440 (30.7)	178 (29.9)	130 (31.6)	127 (25.7)	656 (29.5)	503 (24.2)	n/a	1319 (37.5)	n/a	799 (39.9)	1244 (45.2)	561 (38.1)	595 (21.3)	211 (20.8)	7807 (30.7)	6944 (30.9)
Age 65–75 years	696 (41.3)	231 (40.0)	677 (37.7)	272 (31.4)	3544 (45.1)	2689 (37.6)	1916 (43.1)	937 (34.4)	632 (44.1)	206 (34.6)	155 (37.7)	186 (37.6)	967 (43.4)	794 (38.2)	n/a	1359 (38.6)	n/a	723 (36.1)	1052 (38.3)	540 (36.6)	1113 (39.9)	391 (38.6)	10,752 (42.3)	8328 (37.0)
Age >75 years	408 (24.2)	179 (31.0)	501 (27.9)	320 (37.0)	2236 (28.5)	2658 (37.1)	1066 (24.0)	798 (29.3)	360 (25.1)	212 (35.6)	126 (30.7)	182 (36.8)	604 (27.1)	780 (37.6)	n/a	839 (23.9)	n/a	480 (24.0)	454 (16.5)	373 (25.3)	1080 (38.7)	411 (40.6)	6835 (26.9)	7232 (32.1)
CCI, *n* (%)																								
None	981 (58.2)	347 (60.0)	1106 (61.6)	569 (65.8)	5170 (65.8)	4995 (69.8)	3112 (69.9)	2079 (76.2)	478 (33.4)	274 (46.0)	242 (58.9)	338 (68.3)	1311 (58.9)	1307 (62.9)	n/a	2611 (74.2)	n/a	1513 (75.6)	1710 (62.2)	959 (65.1)	1641 (58.9)	646 (63.8)	15,751 (62.0)	15,638 (69.5)
Moderate	512 (30.4)	175 (30.3)	529 (29.5)	227 (26.2)	2047 (26.1)	1767 (24.7)	1058 (23.8)	563 (20.6)	488 (34.1)	192 (32.2)	114 (27.7)	120 (24.2)	670 (30.1)	616 (29.7)	n/a	744 (21.2)	n/a	410 (20.5)	734 (26.7)	410 (27.8)	882 (31.6)	305 (30.1)	7034 (27.7)	5529 (24.6)
Severe	192 (11.4)	56 (9.7)	160 (8.9)	69 (8.0)	640 (8.1)	398 (5.6)	279 (6.3)	85 (3.1)	466 (32.5)	130 (21.8)	55 (13.4)	37 (7.5)	246 (11.0)	154 (7.4)	n/a	162 (4.6)	n/a	79 (3.9)	306 (11.1)	105 (7.1)	265 (9.5)	62 (6.1)	2609 (10.3)	1337 (5.9)
Marital status, *n* (%)																								
Married/ cohabiting	1051 (62.4)	273 (47.2)	1253 (69.8)	442 (51.1)	5519 (70.2)	3744 (52.3)	3211 (72.2)	1503 (55.1)	851 (59.4)	271 (45.5)	284 (69.1)	261 (52.7)	1517 (68.1)	1029 (49.5)	n/a	2017 (57.4)	n/a	1148 (57.3)	1945 (70.7)	823 (55.8)	1890 (67.8)	476 (47.0)	17,521 (69.0)	11,987 (53.3)
Living alone	634 (37.6)	305 (52.8)	542 (30.2)	423 (48.9)	2338 (29.8)	3416 (47.7)	1238 (27.8)	1224 (44.9)	581 (40.6)	325 (54.5)	127 (30.9)	234 (47.3)	710 (31.9)	1048 (50.5)	n/a	1500 (42.6)	n/a	854 (42.7)	805 (29.3)	651 (44.2)	898 (32.2)	537 (53.0)	7873 (31.0)	10,517 (46.7)
Education, *n* (%)																								
Low	601 (35.7)	298 (51.6)	683 (38.1)	430 (49.7)	2502 (31.8)	3199 (44.7)	1353 (30.4)	1134 (41.6)	564 (39.4)	303 (50.8)	140 (34.1)	237 (47.9)	715 (32.1)	961 (46.3)	n/a	1404 (39.9)	n/a	747 (37.3)	858 (31.2)	623 (42.3)	993 (35.6)	549 (54.2)	8409 (33.1)	9885 (43.9)
Medium	789 (46.8)	177 (30.6)	784 (43.7)	273 (31.6)	3584 (45.6)	2524 (35.3)	2190 (49.2)	991 (36.3)	646 (45.1)	179 (30.0)	165 (40.1)	167 (33.7)	1047 (47.0)	701 (33.8)	n/a	1250 (35.5)	n/a	716 (35.8)	1241 (45.1)	578 (39.2)	1305 (46.8)	344 (34.0)	11,751 (46.3)	7900 (35.1)
High	295 (17.5)	103 (17.8)	328 (18.3)	162 (18.7)	1771 (22.5)	1437 (20.1)	906 (20.4)	602 (22.1)	222 (15.5)	114 (19.1)	106 (25.8)	91 (18.4)	465 (20.9)	415 (20.0)	n/a	863 (24.5)	n/a	539 (26.9)	651 (23.7)	273 (18.5)	490 (17.6)	120 (11.8)	5234 (20.6)	4719 (21.0)

CCI: Charlson Comorbidity Index; *n*: number; n/a: not applicable; SD: standard deviation.

### Contacts to general practice for different abdominal cancers

**Figure 1.**
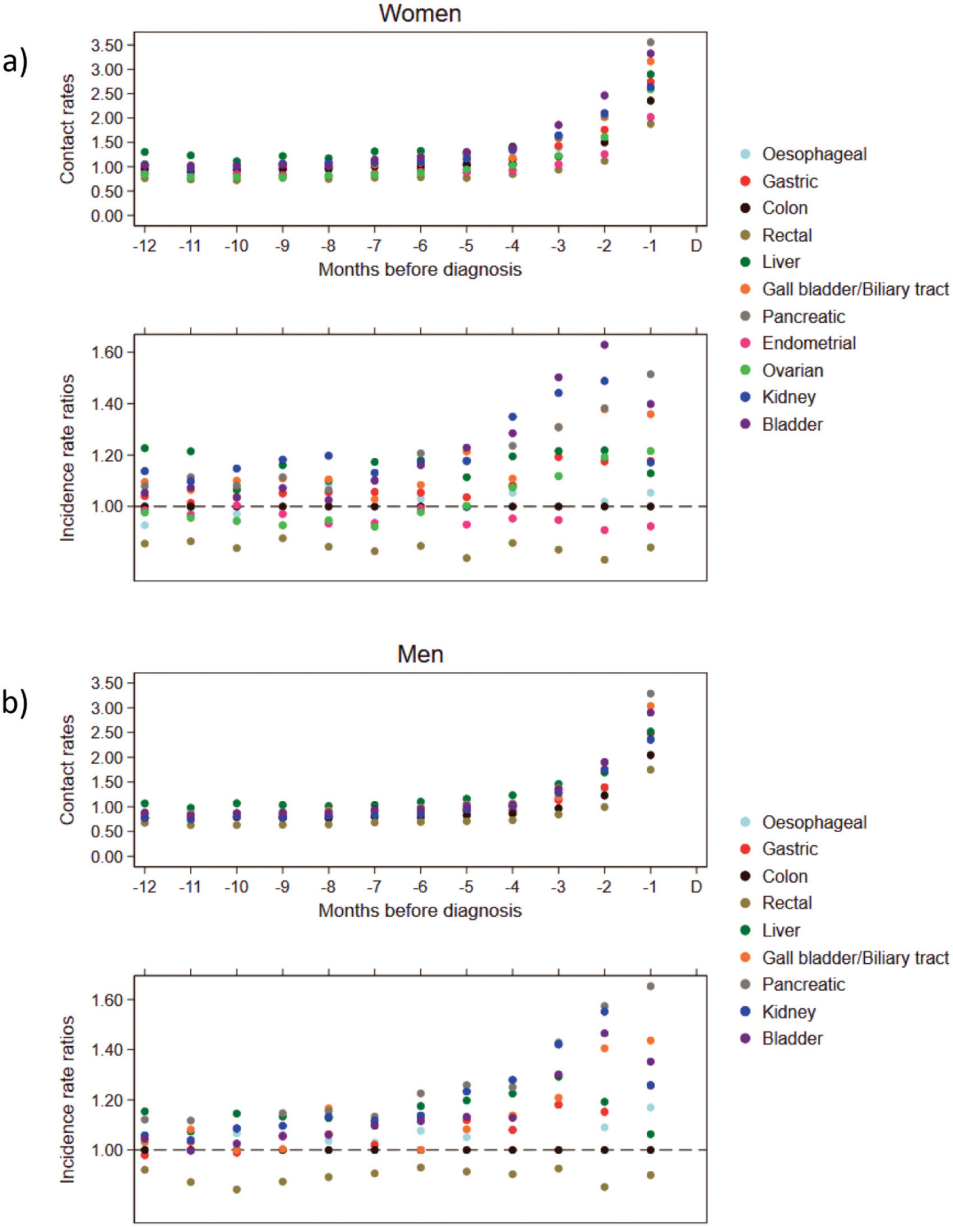
Contacts to general practice across abdominal cancer patients in the 1–12 months preceding the diagnosis. Contacts included daytime face-to-face consultations, telephone consultations and email consultations. Upper part: monthly mean contacts to general practice. Lower part: incidence rate ratios for contacts to general practice compared to colon cancer and adjusted for age, comorbidity, educational level and marital status (95% confidence intervals can be found in Appendix 1). D: date of diagnosis.

A significant increase in contacts to general practice was seen for both sexes from three months prior to the diagnosis across the 11 different abdominal cancer types (Figure 1); however, the contact rates increased at different relative rates (Figure 1). For women with e.g. colon cancer, the mean number of contacts increased from 1.21 (95% CI: 1.17–1.25) in the three months before the diagnosis to 2.36 (95% CI: 2.31–2.41) in the last month before the diagnosis (Figure 1(a)). The results for men demonstrated similar patterns, although men had relatively fewer contacts to general practice than women across all types of abdominal cancers (Figure 1(b)).

Compared to women with colon cancer, women with rectal cancer had fewer contacts to general practice (IRR at 12 months before diagnosis (IRR_–12_)=0.86 (95% CI: 0.80–0.92); IRR at one month before diagnosis (IRR_–1_)=0.85 (95% CI: 0.81–0.89)), whereas women with liver cancer (IRR_–12_=1.23 (95% CI: 1.09–1.38); IRR_–1_=1.11 (95% CI: 1.02–1.20)), pancreatic cancer (IRR_–12_=1.08 (95% CI: 1.01–1.16); IRR_–1_=1.52 (95% CI: 1.45–1.58)) and kidney cancer (IRR_–12_=1.14 (95% CI: 1.05–1.23); IRR_–1_=1.18 (95% CI: 1.12–1.24)) had the highest use of general practice (Figure 1(a) and Appendix 1, Table S1).

During the last 6–7 months before the diagnosis, an increase in contacts to general practice was also seen in bladder cancer patients compared to colon cancer patients, particularly in women, where the greatest IRR was seen at two months preceding the diagnosis (IRR_–2_=1.63 (95% CI: 1.52–1.74)) (Figure 1 and Appendix 1, Table S1).

In both sexes, patients with oesophageal, gastric and gall bladder/biliary tract cancer demonstrated contact rates almost equal to the contact rates for colon cancer (IRR = 1), although an increase in IRR was seen during the last 3–6 months (Figure 1 and Appendix 1, Table S1). Compared to women with colon cancer, women with endometrial cancer had slightly lower contact rates for several months before the diagnosis, while women with ovarian cancer had higher consultation rates during the last three months preceding the diagnosis (Figure 1(a) and Appendix 1, Table S1).

Overall, men showed similar patterns, although the exact estimates were slightly different (Figure 1(b) and Appendix 1, Table S1).

### Contacts to general practice stratified by type of contact

**Figure 2.**
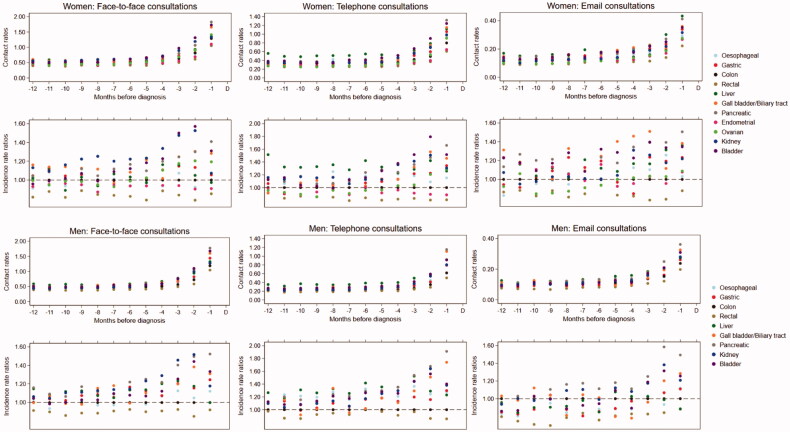
Contacts to general practice across abdominal cancer patients in the 1–12 months preceding the diagnosis stratified by type of contact (daytime face-to-face consultations, telephone consultation and email consultations). Upper part: monthly mean contacts to general practice. Lower part: incidence rate ratios for contacts to general practice compared to colon cancer and adjusted for age, comorbidity, educational level and marital status. 95% confidence intervals can be found in Appendix 1. D: date of diagnosis.

The sub-analyses displayed similar results as the main analyses; the only difference was that patients with liver cancer had a relatively higher proportion of telephone consultations during all 12 months preceding the diagnosis compared to patients with colon cancer (Figure 2, and Appendix 1, Table S2).

## Discussion

### Main findings

In this study of nearly 50,000 first-time abdominal cancer patients, we found a significant increase in contacts to general practice from three months prior to diagnosis. This increase was seen across cancer types and for both sexes. Rectal cancer patients had fewer contacts to general practice than colon cancer patients during all 12 months prior to the diagnosis, whereas patients with liver, pancreatic or kidney cancer had more contacts. A notable increase in contacts to general practice was also found in patients with bladder cancer compared to patients with colon cancer; this increase was particularly seen in women in the last 6–7 months before the diagnosis.

### Strengths and limitations

To our knowledge, this is the first study to compare the frequency and timing of contacts to general practice across different abdominal cancer types in the year preceding a diagnosis of cancer. An important strength was the nationwide design and the inclusion of all incident abdominal cancer patients registered in the DCR during a five-year period. An additional strength was the linkage of this data to other Danish National Registers; these registers are known to generate highly valid and complete data [16], which minimised the risk of selection and information bias.

The main limitation of this study was the lack of information on the reasons for contacting general practice, as such data is not available from the registers. Therefore, some of the contacts might have been unrelated to the cancer disease. However, such contacts would have to vary substantially between the different abdominal cancers to explain the findings of this study, which is unlikely, especially as we included age and comorbidity in the adjusted analyses.

### Comparison with other studies

Our findings of increased use of general practice prior to an abdominal cancer diagnosis is in line with other studies exploring healthcare use for specific cancer types [1,13,14,23]. However, the timing of the increase in contacts to general practice occurred later in our study than in studies comparing cancer patients with references without cancer. Those studies reported increases in contacts for up to 12 months prior to the diagnosis [1,23]. A likely explanation for this discrepancy is that we compared the contact rates between patients with different cancer types. Hence, the mean contact rate is expected to be elevated at several months prior to diagnosis in our population compared to a population of healthy controls. Nevertheless, our finding of increased number of contacts to general practice in the three months before the diagnosis is in line with another Danish study using a similar methodology [14]. Our comparison of contacts for different cancer types makes it possible to gain new knowledge on how different abdominal cancers are diagnosed. This approach highlights the cancers associated with increased use of general practice for a longer period of time before the diagnosis, which can be seen as a proxy for longer diagnostic intervals.

Variations in the proportion of patients who visited their GP three or more times before referral to hospital have been used to categorise cancer types into groups according to diagnostic difficulty [15,24]. According to this classification, colon cancer and oesophageal cancer is categorised as an intermediate-to-diagnose cancer type, while rectal cancer and endometrial cancer belong to the easy-to-diagnose group [15]. This is in line with our findings that rectal cancer patients had lower mean contact rates than colon cancer patients. Additionally, in our study, patients with pancreatic and kidney cancer had higher mean contact rates than colon cancer patients, which is in line with previous studies that categorised these cancers as a hard-to-diagnose cancer types [15]. In contrast, gastric and ovarian cancers are also categorised as hard-to-diagnose cancers [15], and yet we found similar contact rates for these patients and patients with colon cancer in the present study. Still, our results indicated that abdominal cancers characterised as ‘easy’ are those often presenting with bleeding (e.g. rectal and endometrial cancers) while those characterised as ‘hard’ are the solid abdominal tumours (e.g. kidney cancer). However, many cancers have several possible presentations [5,19], which may challenge the clinical utility of this categorisation. Still, our findings suggest that an opportunity exists to detect some abdominal cancers earlier.

Lyratzopoulos et al. also found a strong effect of sex for bladder cancer, including a doubled increased risk in women with bladder cancer to have more than three consultations before a hospital referral [15]. Our findings support this notable increase in the number of contacts to general practice in female bladder cancer patients in the last 6–7 months before a diagnosis. Despite differences in methodologies, the similar findings in both studies suggest that increased attention should be directed towards women presenting repeatedly in general practice, specifically if they present with urinary tract symptoms.

### Clinical implications

Our findings allude to potential missed opportunities across most abdominal cancers. Furthermore, our findings of variations in the use of general practice across the investigated abdominal cancers, suggest that these variations may be caused by different proportions of patients presenting with cancer alarm symptoms across the different abdominal cancer types. This warrants focus on how to improve the diagnostic process and support the GP in the clinical decision-making to improve the chance for more timely diagnosis of cancer. Fast-track cancer patient pathways (CPPs) were implemented in 2007/2008 in Denmark for patients with cancer-alarm symptoms, and followed by a CPP for serious non-specific symptoms and signs in 2012 [6]. However, as most established pathways for cancer diagnosis require specific symptoms or initial GP suspicion of cancer [2,25], a more direct access pathway may be needed for GPs when patients with non-specific or vague symptoms consult [6]. Therefore, novel initiatives aiming to ensure early diagnosis of abdominal cancer may benefit from focussing on development and testing of new referral options for GPs to facilitate the diagnosing of the intermediate- to hard-to-diagnose abdominal cancer types.

Besides better investigation and referral options in general practice, GPs may need clinical decision support tools, such as artificial intelligence (AI) [26] or clearer guidance on effective safety netting for cancer [27]. This could assist the GPs in the initial diagnostic assessment and the diagnostic follow-up. AI could support the GP by recognising and managing cognitive bias to improve the diagnostic accuracy, and safety-netting could help ensure timely and appropriate follow-up when symptoms and signs do not improve [26,27]. Furthermore, point-of-care testing (POCT) is increasingly explored for use in general practice, as POCT may support the early cancer diagnosing in general practice [28–30].

## Conclusions

Increased use of general practice was observed for all abdominal cancers in the months before diagnosis, when examining pre-diagnostic contact rates. The time length of the increase varied across different cancer types. Rectal cancer patients had fewer contacts to general practice during all 12 months preceding the diagnosis compared to colon cancer patients, whereas patients with liver, pancreatic or kidney cancer had more contacts during the 12 months. Thus, abdominal cancers that are hard-to-diagnose tended to cause a larger increase in the number of contacts to general practice over many months before the diagnosis. A notable increase was found in women with bladder cancer compared to women with colon cancer. This implies that increased awareness should be addressed to women presenting with urinary tract symptoms in general practice.

Overall, our findings suggest that opportunities may exist to detect some abdominal cancers earlier. These findings call for additional support to assist GPs in diagnosing abdominal cancer. However, approaches are warranted to overcome the methodological challenges in identifying missed opportunities, particularly for cancers which are not associated with clear ‘alarm’ symptoms.

## Supplementary Material

Supplemental Material

## Data Availability

The datasets included in this study are not publicly available as they contain information that might impair the privacy of the study population. The datasets are stored electronically at Statistics Denmark and can only be accessed by approved collaborators.
