# Supplementary material for: Contacts to general practice in the 12 months preceding a diagnosis of an abdominal cancer: a national register-based cohort study
Source: Scand J Prim Health Care. 2022 Apr 1;40(1):148–56. doi: 10.1080/02813432.2022.2057054 (PMC9090419; doi:10.1080/02813432.2022.2057054)
Supplement: Supplemental Material [file IPRI_A_2057054_SM5496.docx]

Appendix 1.

|  |  |  |  |  |  |  |  |  |  |  |  |  |  |
| --- | --- | --- | --- | --- | --- | --- | --- | --- | --- | --- | --- | --- | --- |
|  |  |  |  |  |  |  |  |  |  |  |  |  |  |
| **Table** **S1.** Incidence rate ratios (with 95% confidence intervals) for general practice consultations in the 1-12 months preceding an abdominal cancer diagnosis compared to colon cancer | | | | | | | |  |  |  |  |  |  |
| **MEN** |  |  |  |  |  |  |  |  |  |  |  |  |  |
| **Cancer type (n)** | **Contacts: total** | -12 | -11 | -10 | -9 | -8 | -7 | -6 | -5 | -4 | -3 | -2 | -1 |
| Oesophageal (1,685) | 21,059 | 1.03(0.95;1.12) | 1.00(0.91;1.08) | 1.07(0.98;1.16) | 1.06(0.97;1.15) | 1.04(0.95;1.13) | 1.03(0.95;1.12) | 1.08(0.99;1.17) | 1.05(0.97;1.14) | 1.08(1.00;1.17) | **1.18(1.10;1.28)** | **1.09(1.02;1.17)** | **1.17(1.11;1.23)** |
| Gastric (1,795) | 22,573 | 0.98(0.90;1.06) | 1.03(0.95;1.12) | 0.99(0.91;1.07) | 1.06(0.97;1.15) | 1.06(0.98;1.16) | 1.02(0.94;1.11) | **1.13(1.04;1.22)** | **1.12(1.04;1.21)** | **1.08(1.00;1.17)** | **1.18(1.10;1.27)** | **1.15(1.08;1.23)** | **1.26(1.20;1.32)** |
| Colon (7,857) | 89,644 | 1.00(1.00;1.00) | 1.00(1.00;1.00) | 1.00(1.00;1.00) | 1.00(1.00;1.00) | 1.00(1.00;1.00) | 1.00(1.00;1.00) | 1.00(1.00;1.00) | 1.00(1.00;1.00) | 1.00(1.00;1.00) | 1.00(1.00;1.00) | 1.00(1.00;1.00) | 1.00(1.00;1.00) |
| Rectal (4,449) | 42,877 | **0.92(0.87;0.98)** | **0.87(0.82;0.93)** | **0.84(0.79;0.90)** | **0.87(0.82;0.93)** | **0.89(0.84;0.95)** | **0.91(0.85;0.96)** | **0.93(0.88;0.99)** | **0.91(0.86;0.97)** | **0.90(0.85;0.96)** | **0.93(0.87;0.98)** | **0.85(0.81;0.90)** | **0.90(0.87;0.94)** |
| Liver (1,432) | 22,029 | **1.15(1.06;1.25)** | **1.07(0.99;1.17)** | **1.15(1.05;1.24)** | **1.13(1.04;1.23)** | **1.13(1.04;1.23)** | **1.10(1.02;1.20)** | **1.18(1.08;1.27)** | **1.20(1.10;1.30)** | **1.23(1.13;1.33)** | **1.29(1.20;1.39)** | **1.19(1.11;1.28)** | 1.06(1.00;1.13) |
| Gall bladder/ biliary tract (411) | 5,735 | 1.03(0.88;1.22) | 1.08(0.93;1.26) | 1.00(0.86;1.16) | 1.00(0.86;1.18) | 1.17(0.99;1.38) | 1.10(0.94;1.29) | 1.00(0.86;1.17) | 1.08(0.94;1.25) | 1.14(0.99;1.31) | **1.21(1.04;1.40)** | **1.41(1.25;1.59)** | **1.44(1.31;1.57)** |
| Pancreatic (2,227) | 33,471 | **1.12(1.04;1.21)** | **1.12(1.04;1.20)** | **1.09(1.01;1.17)** | 1.15(1.07;1.23) | **1.16(1.07;1.24)** | **1.13(1.05;1.22)** | **1.23(1.14;1.31)** | **1.26(1.18;1.35)** | **1.25(1.17;1.34)** | **1.43(1.34;1.52)** | **1.57(1.49;1.67)** | **1.65(1.59;1.73)** |
| Kidney (2,750) | 35,602 | 1.06(0.99;1.14) | 1.04(0.97;1.11) | **1.09(1.01;1.16)** | **1.10(1.02;1.17)** | **1.13(1.06;1.21)** | **1.12(1.04;1.19)** | **1.14(1.06;1.22)** | **1.23(1.15;1.32)** | **1.28(1.20;1.37)** | **1.42(1.34;1.51)** | **1.55(1.47;1.64)** | **1.26(1.20;1.32)** |
| Bladder (2,788) | 40,077 | 1.04(0.98;1.12) | 1.00(0.93;1.07) | 1.03(0.96;1.10) | 1.06(0.99;1.13) | 1.06(0.99;1.13) | **1.10(1.03;1.17)** | **1.12(1.04;1.19)** | **1.13(1.06;1.21)** | **1.13(1.06;1.20)** | **1.30(1.23;1.38)** | **1.47(1.39;1.54)** | **1.35(1.30;1.40)** |
|  |  |  |  |  |  |  |  |  |  |  |  |  |  |
| **WOMEN** |  |  |  |  |  |  |  |  |  |  |  |  |  |
| **Cancer type (n)** | **Contacts: total** | -12 | -11 | -10 | -9 | -8 | -7 | -6 | -5 | -4 | -3 | -2 | -1 |
| Oesophageal (578) | 8,538 | 0.93(0.82;1.05) | 0.99(0.87;1.13) | 0.97(0.86;1.10) | 1.06(0.93;1.20) | 1.08(0.96;1.23) | 1.06(0.94;1.20) | 1.03(0.91;1.16) | 0.99(0.88;1.12) | 1.05(0.93;1.19) | 1.12(1.00;1.25) | 1.02(0.92;1.13) | 1.05(0.99;1.13) |
| Gastric (865) | 13,085 | 1.04(0.94;1.15) | 1.01(0.92;1.12) | 1.04(0.94;1.15) | 1.05(0.95;1.16) | 1.06(0.95;1.17) | 1.06(0.96;1.16) | 1.05(0.95;1.17) | 1.04(0.94;1.15) | 1.08(0.98;1.19) | **1.19(1.09;1.31)** | **1.18(1.09;1.27)** | **1.18(1.10;1.25)** |
| Colon (7,160) | 99,017 | 1.00(1.00;1.00) | 1.00(1.00;1.00) | 1.00(1.00;1.00) | 1.00(1.00;1.00) | 1.00(1.00;1.00) | 1.00(1.00;1.00) | 1.00(1.00;1.00) | 1.00(1.00;1.00) | 1.00(1.00;1.00) | 1.00(1.00;1.00) | 1.00(1.00;1.00) | 1.00(1.00;1.00) |
| Rectal (2,727) | 29,637 | **0.86(0.80;0.92)** | **0.86(0.81;0.93)** | **0.84(0.78;0.90)** | **0.88(0.82;0.94)** | **0.84(0.79;0.90)** | **0.83(0.77;0.88)** | **0.85(0.79;0.91)** | **0.80(0.75;0.86)** | **0.86(0.80;0.92)** | **0.83(0.78;0.89)** | **0.79(0.75;0.84)** | **0.84(0.81;0.88)** |
| Liver (596) | 10,699 | **1.23(1.09;1.38)** | **1.21(1.08;1.37)** | 1.06(0.94;1.20) | **1.16(1.03;1.31)** | 1.10(0.98;1.23) | **1.17(1.05;1.31)** | **1.18(1.04;1.33)** | 1.11(0.98;1.26) | **1.19(1.07;1.33)** | **1.22(1.10;1.34)** | **1.22(1.11;1.34)** | **1.13(1.04;1.22)** |
| Gall bladder/ biliary tract (495) | 8,182 | 1.10(0.96;1.25) | 1.06(0.93;1.21) | 1.10(0.96;1.25) | 1.11(0.97;1.27) | 1.11(0.98;1.25) | 1.03(0.91;1.17) | 1.08(0.96;1.23) | **1.21(1.07;1.38)** | 1.11(0.98;1.25) | **1.31(1.17;1.46)** | **1.38(1.25;1.52)** | **1.36(1.26;1.46)** |
| Pancreatic (2,077) | 35,923 | **1.08(1.00;1.16)** | **1.11(1.04;1.19)** | **1.08(1.00;1.16)** | **1.11(1.04;1.20)** | **1.06(0.99;1.14)** | **1.10(1.03;1.18)** | **1.21(1.13;1.29)** | **1.18(1.10;1.26)** | **1.24(1.16;1.32)** | **1.31(1.23;1.39)** | **1.38(1.31;1.46)** | **1.51(1.45;1.58)** |
| Endometrial (3,517) | 42,521 | 0.99(0.93;1.05) | 0.97(0.91;1.03) | 1.00(0.95;1.07) | 0.97(0.91;1.03) | **0.93(0.88;0.99)** | **0.94(0.88;0.99)** | 0.99(0.93;1.06) | **0.93(0.87;0.99)** | 0.95(0.90;1.01) | 0.95(0.90;1.00) | **0.91(0.86;0.96)** | **0.92(0.89;0.96)** |
| Ovarian (2,002) | 26,356 | 0.98(0.91;1.05) | 0.96(0.89;1.03) | 0.94(0.88;1.02) | 0.93(0.86;1.00) | 0.95(0.88;1.02) | 0.92(0.85;0.99) | 0.98(0.91;1.05) | 1.00(0.93;1.08) | 1.07(1.00;1.15) | **1.12(1.05;1.20)** | **1.19(1.12;1.26)** | **1.22(1.16;1.27)** |
| Kidney (1,474) | 23,956 | **1.14(1.05;1.23)** | **1.10(1.01;1.19)** | **1.15(1.06;1.24)** | **1.18(1.09;1.28)** | **1.20(1.11;1.30)** | **1.13(1.05;1.22)** | **1.17(1.09;1.26)** | **1.18(1.09;1.27)** | **1.35(1.25;1.46)** | **1.44(1.34;1.55)** | **1.49(1.40;1.58)** | **1.17(1.11;1.23)** |
| Bladder (1,013) | 17,988 | 1.05(0.96;1.16) | 1.07(0.97;1.18) | 1.03(0.94;1.14) | 1.07(0.98;1.18) | 1.03(0.93;1.12) | **1.10(1.01;1.21)** | **1.16(1.06;1.27)** | **1.23(1.13;1.34)** | **1.28(1.18;1.40)** | **1.50(1.39;1.63)** | **1.63(1.52;1.74)** | **1.40(1.33;1.47)** |
|  |  |  |  |  |  |  |  |  |  |  |  |  |  |
|  |  |  |  |  |  |  |  |  |  |  |  |  |  |
| **Table** **S2.** Incidence rate ratios (with 95% confidence intervals) for contacts to general practice in the 1-12 months preceding an abdominal cancer diagnosis compared to colon cancer, stratified by type of contact | | | | | | | | |  |  |  |  |  |
| **MEN: Face-to-face consultations** |  |  |  |  |  |  |  |  |  |  |  |  |  |
| **Cancer type (n)** | **Contacts: total** | -12 | -11 | -10 | -9 | -8 | -7 | -6 | -5 | -4 | -3 | -2 | -1 |
| Oesophageal (1,685) | 11,538 | 1.03(0.94;1.14) | 0.93(0.84;1.04) | 1.02(0.92;1.13) | 1.01(0.92;1.12) | 0.97(0.88;1.07) | 0.97(0.88;1.07) | 1.00(0.91;1.09) | 0.98(0.89;1.08) | 1.07(0.97;1.17) | **1.13(1.03;1.23)** | 1.05(0.97;1.13) | **1.12(1.06;1.18)** |
| Gastric (1,795) | 13,277 | 1.00(0.91;1.10) | 1.04(0.94;1.14) | 1.02(0.93;1.12) | 1.08(0.98;1.18) | 1.08(0.99;1.19) | 1.06(0.96;1.16) | **1.17(1.06;1.28)** | **1.13(1.03;1.23)** | 1.07(0.98;1.18) | **1.20(1.11;1.31)** | **1.16(1.08;1.24)** | **1.25(1.18;1.31)** |
| Colon (7,857) | 52,354 | 1.00(1.00;1.00) | 1.00(1.00;1.00) | 1.00(1.00;1.00) | 1.00(1.00;1.00) | 1.00(1.00;1.00) | 1.00(1.00;1.00) | 1.00(1.00;1.00) | 1.00(1.00;1.00) | 1.00(1.00;1.00) | 1.00(1.00;1.00) | 1.00(1.00;1.00) | 1.00(1.00;1.00) |
| Rectal (4,449) | 25,323 | **0.91(0.85;0.98)** | **0.90(0.84;0.97)** | **0.86(0.80;0.92)** | **0.89(0.82;0.95)** | **0.88(0.82;0.95)** | **0.91(0.85;0.97)** | **0.93(0.86;0.99)** | **0.90(0.84;0.96)** | **0.91(0.85;0.97)** | **0.92(0.87;0.99)** | **0.85(0.80;0.90)** | **0.92(0.88;0.96)** |
| Liver (1,432) | 11,904 | **1.15(1.04;1.26)** | 1.08(0.98;1.20) | **1.12(1.01;1.24)** | **1.13(1.02;1.24)** | 1.07(0.97;1.19) | 1.05(0.96;1.15) | **1.13(1.03;1.25)** | **1.14(1.04;1.25)** | **1.20(1.10;1.32)** | **1.23(1.13;1.35)** | **1.18(1.09;1.27)** | 1.00(0.94;1.07) |
| Gall bladder/ biliary tract (411) | 3,268 | 1.00(0.83;1.20) | 1.07(0.89;1.28) | 0.99(0.83;1.18) | 0.98(0.81;1.19) | 1.15(0.93;1.43) | 1.18(0.99;1.42) | 0.99(0.83;1.19) | 1.10(0.94;1.30) | **1.22(1.04;1.42)** | **1.20(1.01;1.43)** | **1.38(1.21;1.58)** | **1.31(1.19;1.44)** |
| Pancreatic (2,227) | 18,815 | **1.16(1.07;1.26)** | **1.09(1.00;1.19)** | **1.07(0.98;1.16)** | **1.17(1.08;1.27)** | **1.11(1.03;1.21)** | **1.09(1.00;1.19)** | **1.22(1.13;1.32)** | **1.25(1.16;1.35)** | **1.22(1.13;1.32)** | **1.40(1.31;1.51)** | **1.50(1.40;1.59)** | **1.52(1.45;1.60)** |
| Kidney (2,750) | 20,346 | 1.06(0.98;1.16) | 1.05(0.96;1.14) | **1.10(1.02;1.20)** | **1.11(1.03;1.20)** | **1.13(1.04;1.22)** | **1.14(1.06;1.23)** | **1.15(1.06;1.24)** | **1.23(1.14;1.33)** | **1.29(1.20;1.39)** | **1.46(1.36;1.56)** | **1.52(1.43;1.61)** | **1.18(1.12;1.24)** |
| Bladder (2,788) | 23,003 | 1.05(0.97;1.13) | 0.98(0.91;1.06) | 1.00(0.93;1.08) | 1.03(0.95;1.11) | 1.03(0.95;1.11) | **1.10(1.02;1.18)** | **1.08(1.00;1.16)** | **1.07(0.99;1.15)** | **1.12(1.04;1.21)** | **1.25(1.17;1.34)** | **1.44(1.36;1.52)** | **1.33(1.28;1.39)** |
|  |  |  |  |  |  |  |  |  |  |  |  |  |  |
| **WOMEN: Face-to-face consultations** |  |  |  |  |  |  |  |  |  |  |  |  |  |
| **Cancer type (n)** | **Contacts: total** | -12 | -11 | -10 | -9 | -8 | -7 | -6 | -5 | -4 | -3 | -2 | -1 |
| Oesophageal (578) | 4,279 | 0.91(0.78;1.07) | 0.94(0.81;1.10) | 0.94(0.81;1.09) | 1.06(0.91;1.23) | 1.01(0.86;1.17) | 1.01(0.87;1.17) | 0.94(0.80;1.10) | 0.97(0.84;1.13) | 0.94(0.80;1.09) | 1.07(0.94;1.23) | 0.93(0.82;1.04) | 1.00(0.93;1.08) |
| Gastric (865) | 6,746 | 1.05(0.93;1.17) | 1.11(0.99;1.26) | 1.04(0.93;1.18) | 1.06(0.94;1.19) | 1.00(0.89;1.12) | 0.99(0.89;1.11) | 1.00(0.89;1.12) | 1.01(0.90;1.14) | 1.11(0.99;1.24) | **1.15(1.03;1.28)** | **1.13(1.04;1.24)** | 1.06(0.98;1.14) |
| Colon (7,160) | 52,652 | 1.00(1.00;1.00) | 1.00(1.00;1.00) | 1.00(1.00;1.00) | 1.00(1.00;1.00) | 1.00(1.00;1.00) | 1.00(1.00;1.00) | 1.00(1.00;1.00) | 1.00(1.00;1.00) | 1.00(1.00;1.00) | 1.00(1.00;1.00) | 1.00(1.00;1.00) | 1.00(1.00;1.00) |
| Rectal (2,727) | 15,965 | **0.82(0.75;0.89)** | **0.88(0.81;0.95)** | **0.82(0.75;0.89)** | **0.89(0.82;0.96)** | **0.85(0.78;0.92)** | **0.84(0.77;0.91)** | **0.83(0.76;0.89)** | **0.79(0.73;0.86)** | **0.89(0.82;0.96)** | **0.84(0.78;0.90)** | **0.79(0.74;0.84)** | **0.85(0.82;0.89)** |
| Liver (596) | 4,929 | 1.02(0.89;1.17) | 1.13(0.99;1.29) | 0.89(0.77;1.03) | 1.03(0.90;1.19) | 0.94(0.82;1.07) | 1.07(0.94;1.21) | 1.03(0.89;1.21) | 0.97(0.83;1.13) | **1.16(1.02;1.33)** | **1.16(1.03;1.31)** | 1.05(0.94;1.17) | 0.97(0.89;1.07) |
| Gall bladder/ biliary tract (495) | 4,304 | 1.16(1.00;1.34) | 1.14(0.98;1.33) | 1.14(0.97;1.34) | 1.13(0.95;1.34) | 1.12(0.97;1.29) | 0.97(0.84;1.12) | 1.08(0.94;1.25) | **1.22(1.06;1.39)** | 1.02(0.88;1.18) | **1.25(1.09;1.43)** | **1.31(1.17;1.46)** | **1.28(1.18;1.40)** |
| Pancreatic (2,077) | 18,351 | 1.04(0.96;1.13) | 1.09(1.00;1.18) | 0.99(0.91;1.08) | **1.12(1.03;1.22)** | **1.05(0.96;1.14)** | **1.12(1.03;1.21)** | **1.17(1.08;1.26)** | **1.10(1.02;1.19)** | **1.20(1.11;1.29)** | **1.25(1.16;1.34)** | **1.30(1.22;1.38)** | **1.41(1.35;1.48)** |
| Endometrial (3,517) | 22,483 | 0.93(0.87;1.00) | 0.95(0.89;1.03) | 0.96(0.90;1.04) | 0.95(0.89;1.02) | **0.87(0.81;0.94)** | 0.95(0.88;1.02) | 0.94(0.87;1.01) | 0.94(0.87;1.01) | 0.94(0.88;1.01) | 0.94(0.88;1.00) | **0.90(0.85;0.96)** | **0.91(0.87;0.95)** |
| Ovarian (2,002) | 14,446 | 0.99(0.91;1.09) | 0.95(0.87;1.04) | 0.99(0.90;1.08) | 0.98(0.90;1.08) | 0.95(0.87;1.04) | 0.93(0.85;1.02) | 0.99(0.91;1.08) | 1.02(0.94;1.11) | **1.11(1.02;1.20)** | **1.18(1.09;1.27)** | **1.20(1.13;1.28)** | **1.19(1.14;1.25)** |
| Kidney (1,474) | 12,979 | **1.13(1.03;1.24)** | 1.10(1.00;1.21) | **1.16(1.06;1.27)** | **1.22(1.12;1.34)** | **1.25(1.14;1.37)** | **1.20(1.10;1.31)** | **1.22(1.12;1.34)** | **1.23(1.13;1.34)** | **1.34(1.22;1.46)** | **1.48(1.37;1.59)** | **1.53(1.43;1.63)** | **1.07(1.01;1.14)** |
| Bladder (1,013) | 9,260 | 0.96(0.86;1.07) | 0.99(0.88;1.11) | 1.02(0.91;1.15) | 1.07(0.96;1.20) | 0.99(0.89;1.10) | 1.09(0.98;1.21) | **1.12(1.01;1.24)** | **1.18(1.07;1.31)** | **1.23(1.11;1.36)** | **1.50(1.37;1.64)** | **1.57(1.46;1.69)** | **1.31(1.24;1.39)** |
|  |  |  |  |  |  |  |  |  |  |  |  |  |  |
|  |  |  |  |  |  |  |  |  |  |  |  |  |  |
| **MEN: Telephone consultations** |  |  |  |  |  |  |  |  |  |  |  |  |  |
| **Cancer type (n)** | **Contacts: total** | -12 | -11 | -10 | -9 | -8 | -7 | -6 | -5 | -4 | -3 | -2 | -1 |
| Oesophageal (1,685) | 7,002 | 1.12(0.99;1.27) | **1.24(1.09;1.40)** | **1.21(1.07;1.38)** | **1.20(1.05;1.36)** | **1.28(1.12;1.45)** | **1.16(1.02;1.31)** | **1.33(1.17;1.50)** | **1.28(1.13;1.45)** | **1.21(1.07;1.36)** | **1.37(1.22;1.53)** | **1.24(1.12;1.38)** | **1.28(1.18;1.39)** |
| Gastric (1,795) | 6,767 | 1.00(0.88;1.14) | **1.13(1.00;1.28)** | 0.99(0.87;1.12) | 1.03(0.90;1.17) | 1.10(0.97;1.25) | 1.05(0.93;1.19) | **1.16(1.02;1.33)** | **1.18(1.05;1.34)** | 1.13(0.99;1.28) | **1.20(1.07;1.34)** | **1.16(1.04;1.29)** | **1.30(1.20;1.40)** |
| Colon (7,857) | 25,583 | 1.00(1.00;1.00) | 1.00(1.00;1.00) | 1.00(1.00;1.00) | 1.00(1.00;1.00) | 1.00(1.00;1.00) | 1.00(1.00;1.00) | 1.00(1.00;1.00) | 1.00(1.00;1.00) | 1.00(1.00;1.00) | 1.00(1.00;1.00) | 1.00(1.00;1.00) | 1.00(1.00;1.00) |
| Rectal (4,449) | 12,548 | 0.98(0.89;1.07) | **0.87(0.79;0.95)** | **0.86(0.78;0.95)** | 0.92(0.84;1.02) | 0.95(0.86;1.05) | 0.92(0.84;1.02) | 1.01(0.92;1.11) | 0.97(0.88;1.07) | 0.91(0.83;1.00) | 0.97(0.88;1.06) | **0.86(0.80;0.94)** | **0.86(0.80;0.91)** |
| Liver (1,432) | 7,503 | **1.27(1.12;1.42)** | **1.18(1.04;1.34)** | **1.31(1.16;1.48)** | **1.26(1.12;1.43)** | **1.33(1.17;1.50)** | **1.26(1.11;1.42)** | **1.42(1.26;1.59)** | **1.36(1.20;1.53)** | **1.35(1.21;1.52)** | **1.53(1.37;1.70)** | **1.29(1.16;1.43)** | **1.23(1.13;1.34)** |
| Gall bladder/ biliary tract (411) | 1,79 | 1.10(0.84;1.43) | 1.15(0.92;1.45) | 0.92(0.73;1.17) | 1.01(0.79;1.29) | **1.34(1.09;1.64)** | 0.95(0.75;1.20) | 1.02(0.80;1.29) | 1.18(0.92;1.52) | 1.11(0.88;1.40) | **1.29(1.06;1.58)** | **1.51(1.27;1.79)** | **1.74(1.53;1.99)** |
| Pancreatic (2,227) | 10,449 | **1.13(1.01;1.26)** | **1.20(1.08;1.34)** | **1.16(1.04;1.29)** | 1.12(1.00;1.25) | **1.24(1.10;1.39)** | **1.21(1.08;1.35)** | **1.29(1.15;1.44)** | **1.30(1.16;1.45)** | **1.35(1.21;1.51)** | **1.54(1.39;1.70)** | **1.68(1.54;1.83)** | **1.91(1.79;2.04)** |
| Kidney (2,750) | 10,774 | 1.10(0.98;1.22) | 1.04(0.93;1.16) | **1.08(0.97;1.20)** | **1.10(0.98;1.22)** | **1.14(1.02;1.26)** | **1.06(0.95;1.18)** | **1.17(1.06;1.30)** | **1.25(1.13;1.39)** | **1.30(1.17;1.43)** | **1.44(1.31;1.58)** | **1.64(1.51;1.78)** | **1.38(1.30;1.48)** |
| Bladder (2,788) | 12,503 | **1.12(1.01;1.24)** | 1.08(0.97;1.20) | 1.08(0.97;1.20) | **1.14(1.02;1.26)** | **1.21(1.09;1.35)** | **1.17(1.06;1.30)** | **1.26(1.14;1.40)** | **1.28(1.15;1.42)** | **1.26(1.14;1.38)** | **1.45(1.32;1.58)** | **1.56(1.44;1.69)** | **1.40(1.32;1.49)** |
|  |  |  |  |  |  |  |  |  |  |  |  |  |  |
| **WOMEN: Telephone consultations** |  |  |  |  |  |  |  |  |  |  |  |  |  |
| **Cancer type (n)** | **Contacts: total** | -12 | -11 | -10 | -9 | -8 | -7 | -6 | -5 | -4 | -3 | -2 | -1 |
| Oesophageal (578) | 3,180 | 0.98(0.83;1.16) | 1.06(0.89;1.27) | 1.02(0.86;1.21) | 1.07(0.90;1.27) | **1.25(1.05;1.48)** | 1.14(0.95;1.35) | 1.18(1.00;1.39) | 1.01(0.86;1.18) | **1.21(1.02;1.44)** | **1.18(1.01;1.39)** | 1.09(0.94;1.26) | **1.15(1.03;1.29)** |
| Gastric (865) | 4,630 | 1.06(0.92;1.23) | 0.90(0.78;1.05) | 1.03(0.88;1.20) | 1.01(0.87;1.17) | 1.05(0.89;1.25) | 1.12(0.97;1.29) | 1.07(0.92;1.25) | 1.10(0.94;1.27) | 1.13(0.98;1.30) | **1.22(1.07;1.39)** | **1.23(1.08;1.39)** | **1.34(1.22;1.47)** |
| Colon (7,160) | 33,823 | 1.00(1.00;1.00) | 1.00(1.00;1.00) | 1.00(1.00;1.00) | 1.00(1.00;1.00) | 1.00(1.00;1.00) | 1.00(1.00;1.00) | 1.00(1.00;1.00) | 1.00(1.00;1.00) | 1.00(1.00;1.00) | 1.00(1.00;1.00) | 1.00(1.00;1.00) | 1.00(1.00;1.00) |
| Rectal (2,727) | 9,913 | **0.91(0.82;1.01)** | **0.83(0.75;0.92)** | **0.87(0.78;0.96)** | **0.85(0.77;0.95)** | **0.85(0.77;0.94)** | **0.80(0.72;0.88)** | **0.84(0.76;0.93)** | **0.80(0.72;0.89)** | **0.82(0.74;0.91)** | **0.83(0.76;0.91)** | **0.81(0.74;0.88)** | **0.81(0.75;0.87)** |
| Liver (596) | 4,281 | **1.52(1.28;1.79)** | **1.32(1.13;1.55)** | **1.32(1.12;1.55)** | **1.33(1.13;1.56)** | **1.36(1.17;1.58)** | **1.28(1.08;1.51)** | **1.42(1.21;1.67)** | **1.32(1.13;1.55)** | **1.23(1.06;1.43)** | **1.29(1.13;1.47)** | **1.42(1.24;1.62)** | **1.26(1.12;1.42)** |
| Gall bladder/ biliary tract (495) | 2,807 | 0.92(0.74;1.13) | 0.91(0.75;1.11) | 1.07(0.90;1.29) | 1.11(0.92;1.35) | 1.04(0.85;1.27) | 1.10(0.93;1.32) | 1.03(0.85;1.24) | 1.14(0.95;1.36) | 1.13(0.94;1.35) | **1.32(1.13;1.54)** | **1.56(1.35;1.81)** | **1.46(1.31;1.63)** |
| Pancreatic (2,077) | 12,831 | **1.12(1.01;1.24)** | 1.09(0.99;1.20) | **1.17(1.06;1.30)** | 1.06(0.95;1.17) | 1.07(0.97;1.18) | 1.10(0.99;1.21) | **1.24(1.12;1.37)** | **1.28(1.16;1.41)** | **1.24(1.13;1.37)** | **1.36(1.24;1.49)** | **1.47(1.36;1.59)** | **1.66(1.56;1.77)** |
| Endometrial (3,517) | 14,043 | 0.99(0.90;1.08) | 0.91(0.83;1.00) | 1.03(0.94;1.12) | 0.93(0.85;1.02) | 0.98(0.89;1.07) | **0.89(0.81;0.98)** | 1.01(0.92;1.10) | 0.92(0.84;1.00) | 0.96(0.88;1.05) | 0.91(0.84;0.99) | **0.89(0.83;0.97)** | **0.89(0.83;0.94)** |
| Ovarian (2,002) | 8,668 | 0.96(0.85;1.07) | 0.93(0.83;1.04) | 0.90(0.80;1.00) | 0.86(0.76;0.96) | 0.96(0.85;1.07) | 0.91(0.81;1.02) | 0.96(0.86;1.07) | 0.96(0.86;1.07) | 1.03(0.92;1.15) | 1.04(0.95;1.15) | **1.21(1.10;1.32)** | **1.29(1.20;1.37)** |
| Kidney (1,474) | 8,188 | **1.16(1.03;1.30)** | 1.13(1.00;1.27) | **1.17(1.04;1.32)** | **1.15(1.03;1.29)** | **1.16(1.03;1.30)** | 1.06(0.94;1.19) | **1.13(1.01;1.26)** | **1.13(1.01;1.26)** | **1.36(1.22;1.52)** | **1.41(1.27;1.57)** | **1.51(1.38;1.65)** | **1.31(1.21;1.41)** |
| Bladder (1,013) | 6,505 | 1.13(0.98;1.29) | **1.16(1.01;1.33)** | 1.04(0.91;1.19) | 1.02(0.90;1.17) | 0.99(0.87;1.13) | 1.12(0.99;1.28) | **1.16(1.01;1.32)** | **1.26(1.12;1.43)** | **1.38(1.22;1.55)** | **1.52(1.36;1.68)** | **1.80(1.63;1.97)** | **1.52(1.40;1.64)** |
|  |  |  |  |  |  |  |  |  |  |  |  |  |  |
|  |  |  |  |  |  |  |  |  |  |  |  |  |  |
| **MEN: Email consultations** |  |  |  |  |  |  |  |  |  |  |  |  |  |
| **Cancer type (n)** | **Contacts: total** | -12 | -11 | -10 | -9 | -8 | -7 | -6 | -5 | -4 | -3 | -2 | -1 |
| Oesophageal (1,685) | 2,519 | 0.83(0.66;1.04) | **0.80(0.65;0.99)** | 0.98(0.80;1.20) | 0.95(0.76;1.19) | 0.84(0.69;1.04) | 1.01(0.82;1.25) | 0.88(0.71;1.09) | 0.88(0.70;1.11) | 0.88(0.72;1.08) | 1.03(0.86;1.25) | 0.93(0.78;1.12) | 1.12(0.96;1.30) |
| Gastric (1,795) | 2,529 | 0.85(0.68;1.07) | 0.83(0.67;1.02) | 0.88(0.72;1.08) | 1.04(0.85;1.27) | 0.88(0.70;1.10) | 0.80(0.65;0.99) | 0.85(0.70;1.05) | 0.89(0.72;1.09) | 0.97(0.79;1.20) | 1.00(0.83;1.21) | 1.07(0.89;1.28) | 1.11(0.96;1.29) |
| Colon (7,857) | 11,707 | 1.00(1.00;1.00) | 1.00(1.00;1.00) | 1.00(1.00;1.00) | 1.00(1.00;1.00) | 1.00(1.00;1.00) | 1.00(1.00;1.00) | 1.00(1.00;1.00) | 1.00(1.00;1.00) | 1.00(1.00;1.00) | 1.00(1.00;1.00) | 1.00(1.00;1.00) | 1.00(1.00;1.00) |
| Rectal (4,449) | 5,006 | **0.80(0.68;0.94)** | **0.75(0.63;0.88)** | **0.71(0.61;0.83)** | **0.70(0.59;0.82)** | **0.79(0.67;0.92)** | **0.84(0.71;0.99)** | **0.76(0.65;0.89)** | **0.81(0.69;0.94)** | **0.83(0.71;0.96)** | **0.83(0.72;0.95)** | **0.84(0.73;0.97)** | **0.88(0.78;0.99)** |
| Liver (1,432) | 2,622 | 0.96(0.77;1.19) | 0.84(0.68;1.05) | 0.90(0.72;1.12) | 0.90(0.73;1.12) | 0.91(0.73;1.15) | 0.98(0.79;1.22) | 0.85(0.68;1.07) | 1.08(0.87;1.33) | 1.03(0.84;1.25) | 1.02(0.84;1.24) | 0.99(0.82;1.20) | 0.89(0.75;1.05) |
| Gall bladder/ biliary tract (411) | 677 | 1.03(0.70;1.51) | 0.98(0.66;1.47) | 1.12(0.77;1.62) | 1.04(0.71;1.53) | 0.81(0.50;1.33) | 1.04(0.68;1.62) | 1.01(0.71;1.45) | 0.80(0.54;1.18) | 0.78(0.52;1.17) | 0.96(0.64;1.44) | 1.20(0.87;1.66) | 1.28(0.97;1.70) |
| Pancreatic (2,227) | 4,207 | 0.94(0.78;1.14) | 1.03(0.85;1.24) | 1.01(0.85;1.22) | 1.10(0.92;1.33) | 1.16(0.97;1.39) | 1.17(0.98;1.40) | 1.11(0.93;1.33) | 1.18(0.99;1.40) | 1.11(0.94;1.31) | **1.25(1.07;1.47)** | **1.59(1.37;1.83)** | **1.49(1.31;1.70)** |
| Kidney (2,750) | 4,482 | 0.94(0.78;1.12) | 1.00(0.84;1.20) | 0.98(0.82;1.17) | 0.99(0.83;1.19) | 1.09(0.92;1.30) | 1.10(0.93;1.31) | 0.99(0.84;1.17) | 1.12(0.95;1.32) | 1.08(0.92;1.27) | **1.17(1.01;1.37)** | **1.38(1.20;1.59)** | **1.21(1.07;1.37)** |
| Bladder (2,788) | 4,571 | 0.86(0.71;1.03) | 0.87(0.72;1.04) | 1.03(0.87;1.22) | 1.00(0.83;1.19) | 0.89(0.74;1.07) | 0.92(0.77;1.11) | 0.94(0.80;1.11) | 1.09(0.93;1.29) | 0.89(0.76;1.05) | **1.19(1.03;1.38)** | **1.31(1.15;1.51)** | **1.26(1.12;1.41)** |
|  |  |  |  |  |  |  |  |  |  |  |  |  |  |
| **WOMEN: Email consultations** |  |  |  |  |  |  |  |  |  |  |  |  |  |
| **Cancer type (n)** | **Contacts: total** | -12 | -11 | -10 | -9 | -8 | -7 | -6 | -5 | -4 | -3 | -2 | -1 |
| Oesophageal (578) | 1,079 | 0.83(0.60;1.14) | 1.00(0.73;1.37) | 0.95(0.69;1.31) | 0.99(0.71;1.38) | 0.95(0.69;1.30) | 1.00(0.73;1.35) | 0.99(0.74;1.33) | 1.08(0.82;1.43) | 1.12(0.85;1.48) | 1.10(0.82;1.48) | 1.26(0.98;1.62) | 1.03(0.84;1.27) |
| Gastric (865) | 1,709 | 0.94(0.73;1.23) | 0.91(0.70;1.20) | 1.03(0.79;1.33) | 1.09(0.85;1.40) | 1.23(0.98;1.56) | 1.13(0.88;1.44) | 1.20(0.95;1.51) | 0.97(0.76;1.24) | 0.85(0.66;1.09) | 1.24(0.98;1.55) | 1.19(0.97;1.47) | **1.23(1.03;1.47)** |
| Colon (7,160) | 12,542 | 1.00(1.00;1.00) | 1.00(1.00;1.00) | 1.00(1.00;1.00) | 1.00(1.00;1.00) | 1.00(1.00;1.00) | 1.00(1.00;1.00) | 1.00(1.00;1.00) | 1.00(1.00;1.00) | 1.00(1.00;1.00) | 1.00(1.00;1.00) | 1.00(1.00;1.00) | 1.00(1.00;1.00) |
| Rectal (2,727) | 3,759 | 0.87(0.72;1.05) | 0.88(0.73;1.07) | 0.82(0.68;1.00) | 0.88(0.73;1.06) | **0.81(0.68;0.97)** | 0.85(0.71;1.01) | 0.94(0.79;1.12) | **0.83(0.70;0.99)** | **0.82(0.70;0.97)** | **0.78(0.66;0.92)** | **0.79(0.68;0.92)** | 0.88(0.77;1.00) |
| Liver (596) | 1,489 | 1.23(0.91;1.65) | 1.16(0.86;1.57) | 1.04(0.77;1.39) | 1.12(0.84;1.50) | 1.02(0.76;1.36) | **1.21(0.93;1.58)** | 1.12(0.83;1.52) | 1.09(0.79;1.49) | 1.17(0.89;1.52) | 1.17(0.91;1.50) | **1.31(1.03;1.67)** | **1.36(1.12;1.66)** |
| Gall bladder/ biliary tract (495) | 1,071 | 1.31(0.94;1.83) | 1.18(0.80;1.76) | 0.98(0.69;1.38) | 1.00(0.74;1.37) | 1.33(0.96;1.83) | 1.09(0.78;1.53) | 1.25(0.91;1.71) | **1.40(1.03;1.92)** | **1.46(1.09;1.96)** | **1.51(1.13;2.03)** | 1.12(0.85;1.47) | **1.38(1.09;1.75)** |
| Pancreatic (2,077) | 4,741 | 1.13(0.93;1.38) | **1.27(1.06;1.52)** | 1.20(1.00;1.45) | 1.21(1.00;1.47) | 1.06(0.88;1.28) | 1.01(0.84;1.21) | **1.24(1.04;1.47)** | 1.18(0.99;1.39) | **1.34(1.12;1.61)** | **1.39(1.18;1.65)** | **1.39(1.20;1.62)** | **1.51(1.32;1.71)** |
| Endometrial (3,517) | 5,995 | **1.23(1.06;1.44)** | 1.17(1.00;1.36) | 1.11(0.95;1.29) | 1.13(0.97;1.32) | 1.05(0.90;1.23) | 0.99(0.84;1.16) | 1.16(1.00;1.35) | 0.93(0.80;1.08) | 0.96(0.82;1.11) | 1.03(0.89;1.19) | 0.96(0.84;1.09) | 1.08(0.96;1.21) |
| Ovarian (2,002) | 3,242 | 0.92(0.75;1.13) | 1.06(0.87;1.29) | 0.85(0.69;1.03) | 0.85(0.70;1.04) | 0.87(0.72;1.06) | 0.91(0.75;1.11) | 0.94(0.77;1.14) | 1.00(0.83;1.20) | 1.02(0.85;1.21) | 1.03(0.87;1.23) | 1.03(0.88;1.21) | 1.09(0.94;1.26) |
| Kidney (1,474) | 2,789 | 1.07(0.86;1.35) | 0.95(0.75;1.21) | 0.98(0.78;1.22) | 1.03(0.83;1.28) | 1.03(0.83;1.28) | 1.01(0.82;1.24) | 1.01(0.82;1.23) | 1.04(0.85;1.28) | **1.31(1.08;1.59)** | **1.26(1.03;1.54)** | 1.18(0.99;1.40) | **1.22(1.04;1.43)** |
| Bladder (1,013) | 2,223 | 1.23(0.97;1.55) | 1.18(0.92;1.52) | 1.09(0.86;1.39) | 1.17(0.92;1.49) | **1.28(1.03;1.60)** | 1.08(0.86;1.37) | **1.32(1.06;1.65)** | **1.29(1.04;1.59)** | 1.23(0.99;1.53) | **1.40(1.15;1.70)** | **1.34(1.10;1.63)** | **1.35(1.15;1.58)** |
|  |  |  |  |  |  |  |  |  |  |  |  |  |  |
